# Supplementary material for: Recovery of Naringin-Rich Flavonoid Extracts from Agroresidues with Anxiolytic- and Antidepressant-like Effects in Mice
Source: Molecules. 2022 Dec 3;27(23):8507. doi: 10.3390/molecules27238507 (PMC9740236; doi:10.3390/molecules27238507)
Supplement: Supplementary file 1 [file molecules-27-08507-s001.zip › molecules-2015317-SI.pdf]

Supplementary Material

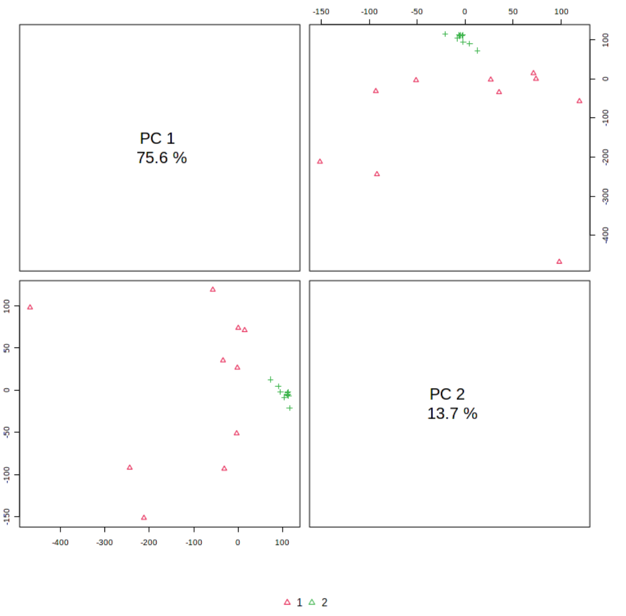

Figure S1 PC1 and PC2 score plot from ^{1}H NMR, red triangles corresponding to methanolic fraction and green to aqueous fraction, explained variance is shown in the corresponding diagonal cell.

Table S1 Effects of the methanol extract (M1), imipramine (IMI), and fluoxetine (FLX) on the ambulatory activity in the Open Field Test.

Acute treatment	Doses (mg/kg)	Rearings number	Count number
CTL	0	33.6±1.3	45.0±2.3
M1	25	30.1±1.8	44.0±2.1
	50	27.6±2.1	41.7±2.2
	100	29.7±0.9	40.0±2.4
	200	29.1±2.4	41.8±2.7
	400	29.2±1.8	41.8±2.7
IMI	25	29.5±1.33	40.7±2.4
		H= 5.99, fd= 6, p= 0.42	H= 2.3, fd= 6, p= 0.88
Repeated treatment (7days)			
	0	28.3±2.5	42.7±4.6
	25	27.0±1.8	46.33.0
	50	29.0±3.1	45.2±4.4
	100	25.0±0.8	42.2±2.8
	200	27.3±2.8	39.6±3.4
FLX 5	5	28.2±1.5	49.12±4.6
		H= 2.31, fd= 5, p=0.80	H= 4.369, fd=5, p= 0.49

Table S2 Effect of diazepam (DZ) on the ethological behaviors on the Elevated Plus Maze Test (EPM). * p≤ 0.05, ** p≤ 0.01, *** p ≤ 0.001

Treatment/dose (mg/kg)	CPT (s) mean± sem	head dipping number (mean± sem)	stretches number (mean± sem)
CTL	82.8 ±6.1	15.7±0.8	31.8±1.9
25	83.4±9.2	20.7±1.5*	23.8±1.8
50	77.7±12.8	30.7±2.1***	18.0±1.0***
100	64.1±1.1	30.2±2.3***	21.3±1.1***
200	87.0±10.09	17.7±1.3	24.6±0.8***
400	69.3±5.6	13.7±1.0	27.7±1.8
	H=6.4, fd=5, p=0.26	H=32.3, fd=5, p≤0.001	H=25.2, fd=5, p≤0.001
CTL	66.9±5.1	16.7 ±0.6	24.3±1.7
DZ 0.25	72.1±5.8	12.2 ±0.7+	24.1±1.6
DZ 0.5	94.7±8.7**	21.3 ±1.0**	21.0±1.1
DZ 1.0	85.0±8.7	26.2 ±0.9***	14.1±0.6**
DZ 2.0	55.5±5.5	35.6 ±1.1***	12.5±0.5***
	H = 23.6, fd= 4, p≤.001	H = 41.7, fd= 4, p≤0.001	H = 32.6, fd=4, p≤0.001
